# Identification of differentially expressed genes in the development of osteosarcoma using RNA-seq

**DOI:** 10.18632/oncotarget.13554

**Published:** 2016-11-24

**Authors:** Yihao Yang, Ya Zhang, Xin Qu, Junfeng Xia, Dongqi Li, Xiaojuan Li, Yu Wang, Zewei He, Su Li, Yonghong Zhou, Lin Xie, Zuozhang Yang

**Affiliations:** ^1^ Bone and Soft Tissue Tumors Research Center of Yunnan Province, Department of Orthopaedics, The Third Affiliated Hospital of Kunming Medical University (Tumor Hospital of Yunnan Province), Kunming, Yunnan 650118, China

**Keywords:** differentially expressed genes, network, primary osteosarcoma, metastatic osteosarcoma, target

## Abstract

**Objective:**

Osteosarcoma (OS) is a malignant bone tumor with high morbidity in young adults and adolescents. This study aimed to discover potential early diagnosis biomarkers in OS.

**Results:**

In total, 111 differentially expressed genes (DEGs) were identified in primary OS compared with normal controls and 235 DEGs were identified in metastatic OS compared with primary OS. AURKB and PPP2R2B were the significantly up-regulated and down-regulated hub proteins, respectively, in the PPI protein-protein network (PPI) network of primary OS. ISG15 and BTRC were the significantly up-regulated and down-regulated hub proteins, respectively, in the network of metastatic OS. The DEGs in metastatic OS compared with primary OS were significantly enriched in the arachidonic acid metabolism, malaria, and chemokine signaling pathways. Finally, we employed quantitative real-time polymerase chain reaction (qRT-PCR) to validate the expression levels of candidate DEGs and the results indicated that our bioinformatics approach was acceptable.

**Materials and Methods:**

The mRNA expression profiling of 20 subjects was obtained through high-throughput RNA-sequencing. DEGs were identified between primary OS and normal Control, and between primary OS and metastatic OS, respectively. Functional annotation and PPI networks were used to obtain insights into the functions of DEGs. qRT-PCR was performed to detect the expression levels of dysregulated genes in OS.

**Conclusions:**

Our work might provide groundwork for the further exploration of tumorigenesis and metastasis mechanisms of OS.

## INTRODUCTION

Osteosarcoma (OS) is a common primary malignant bone tumor with high morbidity in young adults and adolescents [1]. The most prevalent locations for OS are the distal femur and proximal tibial metaphyses [2]. OS is characterized by high local aggressiveness and rapid-metastasizing potential to the lungs and results in poor survival for patients with OS.

The therapeutic strategies for OS include wide tumor resection, adjuvant chemotherapy, and radiotherapy, which have significantly improved the prognosis of patients with malignancy [3]. Despite extensive advancements in surgical techniques, the 5-year survival rate of OS patients remains at 60–70% [4]. Lung metastasis contributes to the primary cause of mortality in OS patients, and the 5-year survival rate of patients with metastatic OS is only 10 to 20% [5].

To date, the pathogenesis of OS is not elucidated. It is reported that miRNAs, cytokines, dysregulated genes, and gene polymorphisms are associated with OS tumorigenesis and metastasis. miR-542-5p plays a critical role in cell proliferation and promotes OS tumorigenesis by targeting HUWE1, which predicts a poor prognosis for OS patients [6]. Decreased IL-6 expression inhibits OS growth and metastasis via activation of the STAT3 and ERK pathways *in vitro* and in the nude mouse model. In addition, IL-6 suppression reduces tumor self-seeding by circulating tumor cells in OS [7]. High expression of Gelsolin (GSN) correlates with tumor size, advanced Enneking stage, and poor prognosis in OS patients. Knockdown of GSN significantly inhibits cell proliferation and invasiveness through the down-regulation of p-AKT and the p-P38 pathway [8]. In the Chinese Han population, IL-10 -1082A/G and IL-8 -251 A/T genotypes are associated with increased susceptibility of OS, and the IL-8 -251 A/T genotype increases risk for development and metastasis in patients with OS [9, 10].

OS, with high malignancy and metastasizing potential to the lungs frequently results in poor survival. Therefore, the study of biomarkers for OS development and metastasis is important for improving the survival of patients.

In this study, we used high-throughput RNA-sequencing to obtain mRNA expression data from peripheral blood of normal controls, primary OS and metastatic OS patients and to identify differentially expressed genes between primary OS and normal control and between metastatic OS and primary OS. Our aim was to provide valuable information for the identification of early diagnosis biomarker for OS development and metastasis.

## RESULTS

### Transcriptome sequencing of subjects

High throughput RNA-sequencing was performed on the blood samples of 10 subjects with OS (5 primary OS patients and 5 metastatic OS patients) and 10 healthy controls. Control samples were pooled for RNA-seq. Approximately, 1.7 × 10^7^, 1.7 × 10^7^, 1.8× 10^7^, 2.8 × 10^7^, and 3.1 × 10^7^ sequencing reads were generated from metastatic OS specimens; 2.7× 10^7^, 1.4× 10^7^, 2.5× 10^7^, 1.5× 10^7^, and 2.9× 10^7^ reads were generated from primary OS specimens; and 1.4 × 10^7^ reads were generated from pooled normal specimens, as Table 1 shows. All of the sequencing reads were aligned to the UCSC human reference genome (hg.19). Basic information about the patients is shown in Supplementary Table S1.

**Table 1:** Transcriptome sequencing of subjects

Sample_ID	Total_Reads	Total_Bases	Error%
M1	16968330	2562217830	0.0164
M2	16945590	2558784090	0.0147
M3	18392754	2777305854	0.0149
M4	27963602	4222503902	0.0200
M5	31299610	4726241110	0.0150
P1	27065992	4086964792	0.0155
P2	14319834	2162294934	0.0169
P3	24920680	3763022680	0.0187
P4	15312548	2312194748	0.0154
P5	28589860	4317068860	0.0149
HC	14359676	2020949388	0.0131

M: metastatic OS; P: primary OS; HC: healthy control

### DEGs between primary osteosarcoma and healthy control

The mRNA expression data of the 5 primary OS blood samples and 1 pooled normal blood sample was obtained using RNA-seq. In total, 111 significantly DEGs were identified in primary OS compared with normal control, which consisted of 68 up- and 43 down-regulated genes. CD177 was the most significantly up-regulated gene and CMKT2 was the most significantly down-regulated gene. The top 15 genes exhibiting significant up- and down-regulation are listed in Table 2. The full list of DEGs is shown in Supplementary Table S2.

**Table 2:** The DEGs in primary osteosarcoma compared to the normal control

Gene ID	Gene Symbol	*P*-value	log^2^FC
**Up-regulation genes (top 15)**			
57126	CD177	0.00365	7.28443
131540	ZDHHC19	0.0185	6.988027
10926	DBF4	0.0307	5.21743
387755	INSC	0.02145	4.575003
101928079	LINC01057	0.0209	3.910375
56729	RETN	0.04225	3.466679
6507	SLC1A3	0.0346	3.424351
1958	EGR1	0.00105	3.334501
202051	SPATA24	0.0277	3.273086
84418	CYSTM1	0.0357	3.258481
4651	MYO10	0.0489	3.058558
79623	GALNT14	0.0489	3.024365
6129	RPL7	0.00315	2.909688
50486	G0S2	0.0291	2.894525
3622	ING2	0.04375	2.81313
**Down-regulation genes (top 15)**			
1160	CKMT2	0.01115	−4.28207
1088	CEACAM8	0.01075	−3.24111
10964	IFI44L	0.00465	−3.14574
140462	ASB9	0.0327	−2.73412
91543	RSAD2	0.0133	−2.60049
653519	GPR89A	0.0213	−2.5051
55183	RIF1	0.04975	−2.49066
7813	EVI5	0.02925	−2.41565
84725	PLEKHA8	0.02415	−2.39095
4057	LTF	0.0033	−2.30934
79828	METTL8	0.02985	−2.28365
79750	ZNF385D	0.02565	−2.19376
653464	SRGAP2C	0.0349	−2.10654
643699	GOLGA8N	0.04365	−2.10211
23230	VPS13A	0.0419	−2.07423

FC: fold change.

### Functional annotation of DEGs between primary osteosarcoma and healthy control

GO annotation was performed using the 111 DEGs in primary OS to obtain insights into their biological roles. The threshold for GO terms was *P* < 0.05. Regulation of microtubule polymerization or depolymerization (GO: 0031110), post-embryonic development (GO:0009791), and regulation of ossification (GO: 0030278) were the most enriched biological processes. Protein binding (GO: 0005515), microtubule plus-end binding (GO: 0051010), and voltage-gated calcium channel activity (GO: 0005245) were the most enriched molecular functions. Cytoplasm (GO: 0005737), plasma membrane (GO: 0005886), and cytosol (GO: 00058293) were the most enriched cellular components. These data are shown in Supplementary Table S3. The signaling pathways of DEGs between primary OS and normal controls were not available in the KEGG database.

### PPI networks of DEGs between primary OS and healthy control

**Figure 1:**
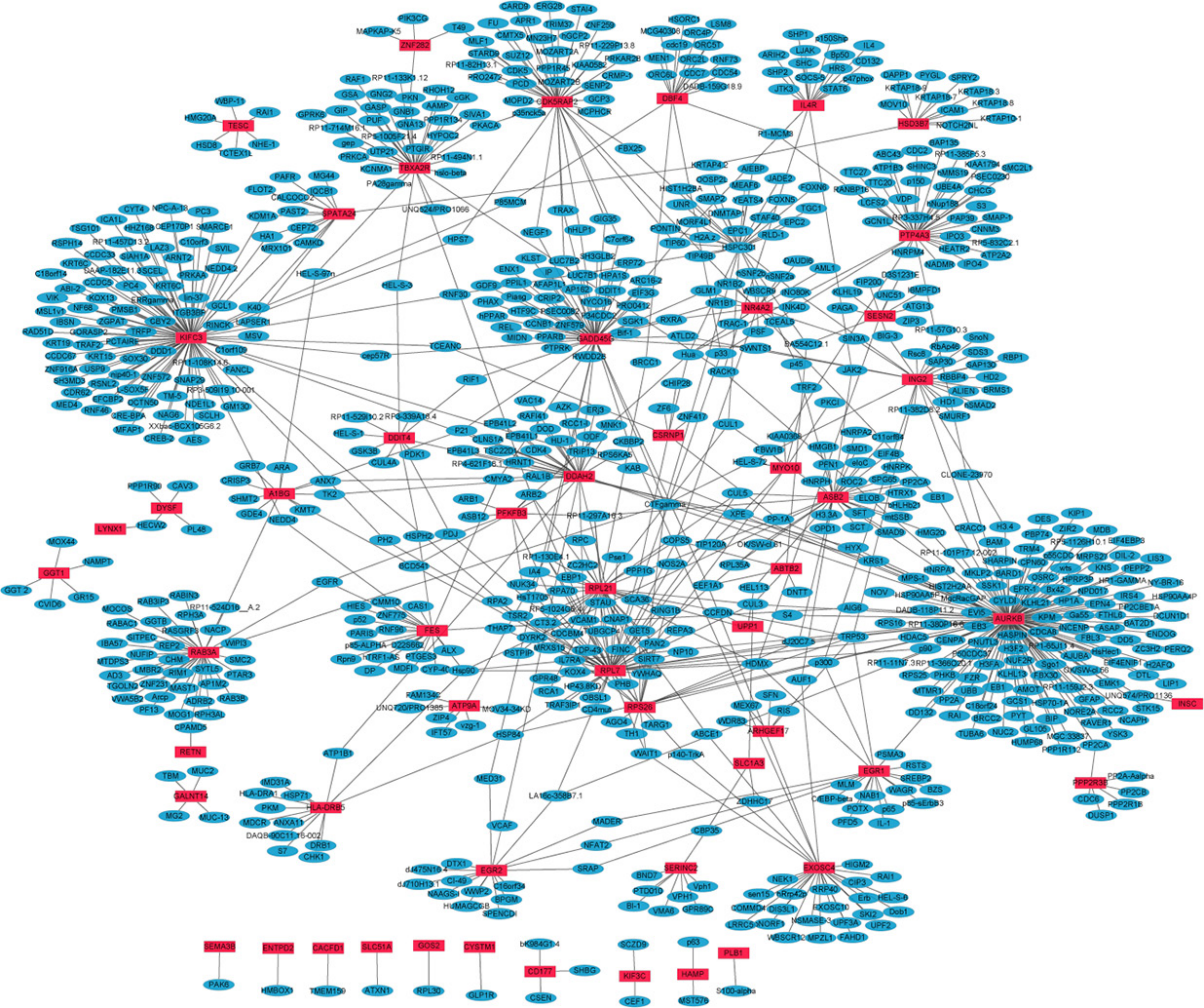
The constructed PPI network of up-regulated DEGs between primary OS and normal control The red nodes represent up-regulated DEGs and the blue nodes denote gene products predicted to interact with the DEGs.

PPI networks of 68 up- and 43 down-regulated DEGs in primary OS were constructed by Cytoscape. Hub proteins indicated that the nodes had high connectivity with other nodes. In the up-regulated DEGs network, 835 nodes and 964 edges were available and the most significantly up-regulated hub protein was AURKB (Figure 1). In the down-regulated DEGs network, 579 nodes and 597 edges were available and the most significantly down-regulated hub protein was SMN1 (Supplementary Figure S1).

### DEGs between primary OS and metastatic OS

RNA-seq was also used to obtain mRNA expression data of the 5 metastatic OS patients. In total, 235 significant DEGs were identified in metastatic OS compared with primary OS, which consisted of 109 up- and 126 down-regulated genes. ALAS2 was the most significantly up-regulated gene and ZDHHC19 was the most significant down-regulated gene. The top 15 genes exhibiting significant up- and down-regulation are listed in Table 3. The full list of DEGs is shown in Supplementary Table S4.

**Table 3:** The DEGs between primary osteosarcoma and metastatic osteosarcoma

Gene ID	Gene Symbol	*P*-value	log_2_FC
**Up-regulation genes (top 15)**			
212	ALAS2	0.00135	4.412527
759	CA1	5.00E–05	4.367439
3045	HBD	0.0238	3.778141
3048	HBG2	0.0045	3.703257
8991	SELENBP1	0.0145	3.531732
7262	PHLDA2	0.00505	3.492722
1991	ELANE	5.00E–05	3.1807
4353	MPO	5.00E–05	3.001248
4680	CEACAM6	6.00E–04	2.974953
27285	TEKT2	0.0261	2.965083
3397	ID1	4.00E–04	2.955801
116028	RMI2	0.0043	2.895913
105	ADARB2	0.0061	2.891606
1669	DEFA4	5.00E–05	2.888638
1088	CEACAM8	1.00E–04	2.82414
**Down-regulation genes (top 15)**			
131540	ZDHHC19	5.00E–05	–5.74283
57126	CD177	5.00E–05	–5.31558
79071	ELOVL6	0.0075	–3.58835
6507	SLC1A3	1.00E–04	–3.28638
2829	XCR1	0.0041	–2.88172
84976	DISP1	1.00E–04	–2.71269
1.02E+08	LINC01057	0.00265	–2.58354
3248	HPGD	0.00455	–2.47742
125965	COX6B2	0.01395	–2.40323
2258	FGF13	0.01515	–2.39481
7060	THBS4	0.01375	–2.39479
8945	BTRC	1.00E–04	–2.3576
146433	IL34	0.0235	–2.30004
122402	TDRD9	0.0024	–2.28361
10079	ATP9A	0.0023	–2.17761

FC: fold change.

### Functional analysis of DEGs between primary OS and metastatic OS

GO annotation was performed using the 235 DEGs between primary OS and metastatic OS. The threshold for GO terms was FDR < 0.05. Cytokine-mediated signaling pathway (GO: 0019221), type I interferon-mediated signaling pathway (GO: 0060337), and immune response (GO: 0006955) were the most enriched biological processes. Cytoplasm (GO: 0005737), plasma membrane (GO: 0005886), and membrane (GO: 0016020) were the most enriched cellular components. Protein binding (GO: 0005515), metal ion binding (GO: 0046872) and receptor activity (GO: 0004872) were the most enriched molecular functions. These data are shown in Supplementary Table S5.

We performed the KEGG pathway enrichment analysis for DEGs with a threshold FDR value < 0.05. Pathways with the greatest enrichment were arachidonic acid metabolism (hsa00590), malaria (hsa05144) and chemokine signaling pathway (hsa04062), as Supplementary Table S6 shows.

### PPI networks of DEGs between primary OS and metastatic OS

**Figure 2:**
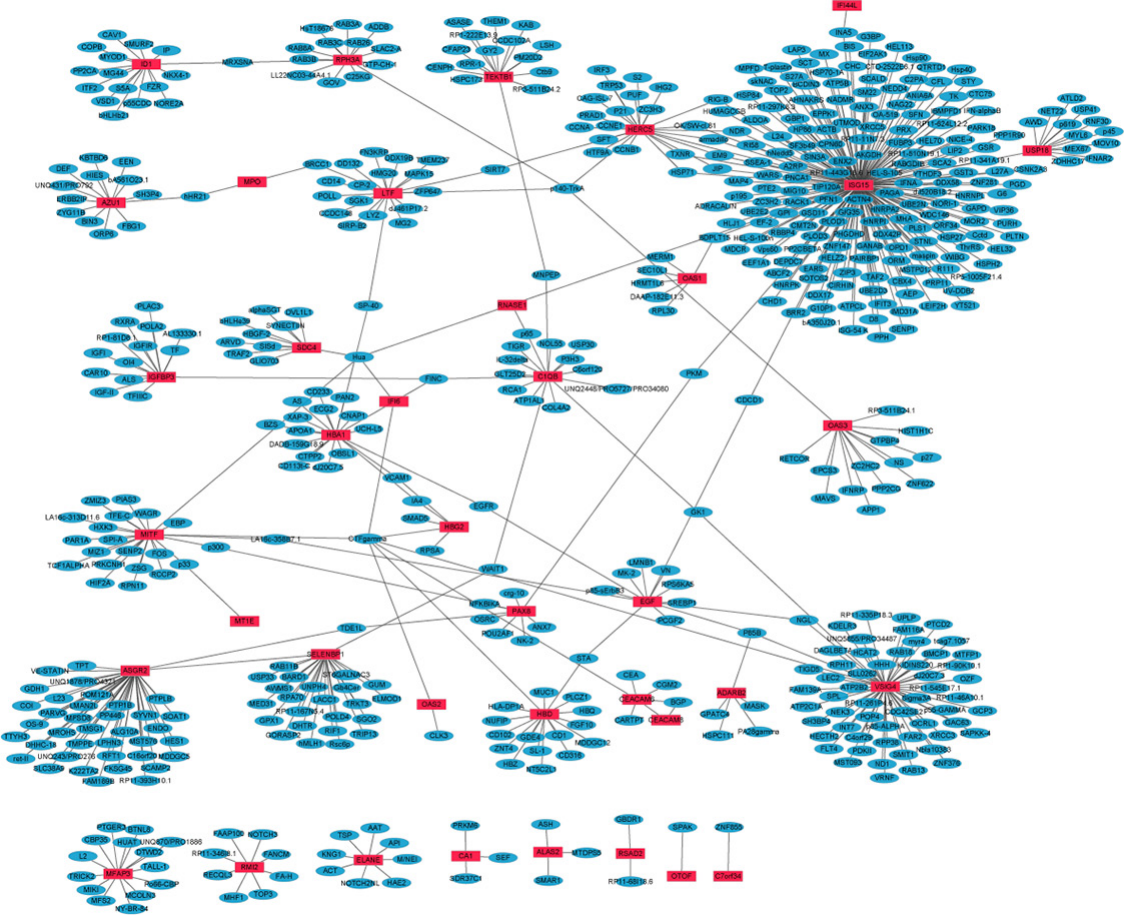
The constructed PPI network of the top 50 up-regulated DEGs between metastatic OS and primary OS The red nodes represent up-regulated DEGs and the blue nodes denote gene products predicted to interact with the DEGs.

PPI networks of the top 50 up-regulated and top 50 down-regulated DEGs between primary OS and metastatic OS were constructed by Cytoscape. In the up-regulated DEGs network, 617 nodes and 629 edges were available and the most significantly up-regulated hub protein was ISG15 (Figure 2). In the down-regulated DEGs network, 505 nodes and 511 edges were available and the most significantly down-regulated hub protein was BTRC (Supplementary Figure S2).

### Verification of the expression level of DEGs between primary OS and healthy control

**Figure 3:**
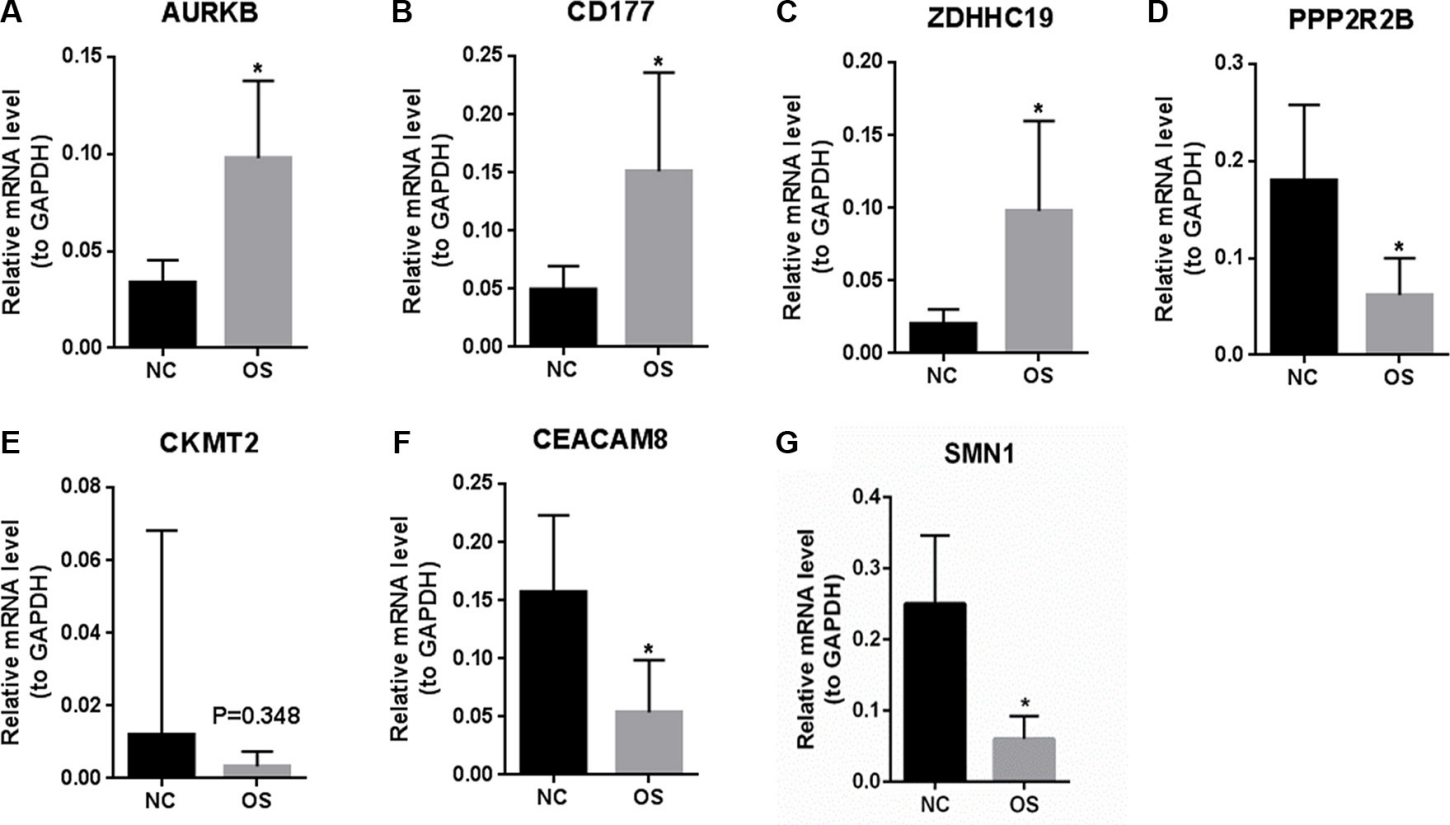
The expression levels of DEGs between primary OS and healthy controls were verified by qRT-PCR (**A**): AURKB; (**B**): CD177; (**C**): ZDHHC19; (**D**): PPP2R2B; (**E**): CKMT2; (**F**): CEACAM8; (**G**): SMN1. NC represents tissues of normal control and OS represents osteosarcoma. At least three independent experiments were performed for statistical evaluation.

To verify the RNA-seq analysis data, the expression levels of DEGs of 14 normal tissues and 19 OS samples without metastasis (named as primary group) were quantified by qRT-PCR. As Figure 3A, and 3C shown, AURKB, CD177 and ZDHHC19 were significantly up-regulated in primary OS compared with normal controls (*P* < 0.05). In Figure 3D, and 3G, PPP2R2B, CEACAM8 and SMN1 were significantly down-regulated in primary OS compared with normal controls (*P* < 0.05). The expression level of CMKT2 had no significant difference between primary OS and normal controls but showed a tendency for down-regulation in primary OS. The qRT-PCR results between primary OS and healthy controls verified our RNA-seq data.

### Verification of the expression level of DEGs between primary OS and metastatic OS

**Figure 4:**
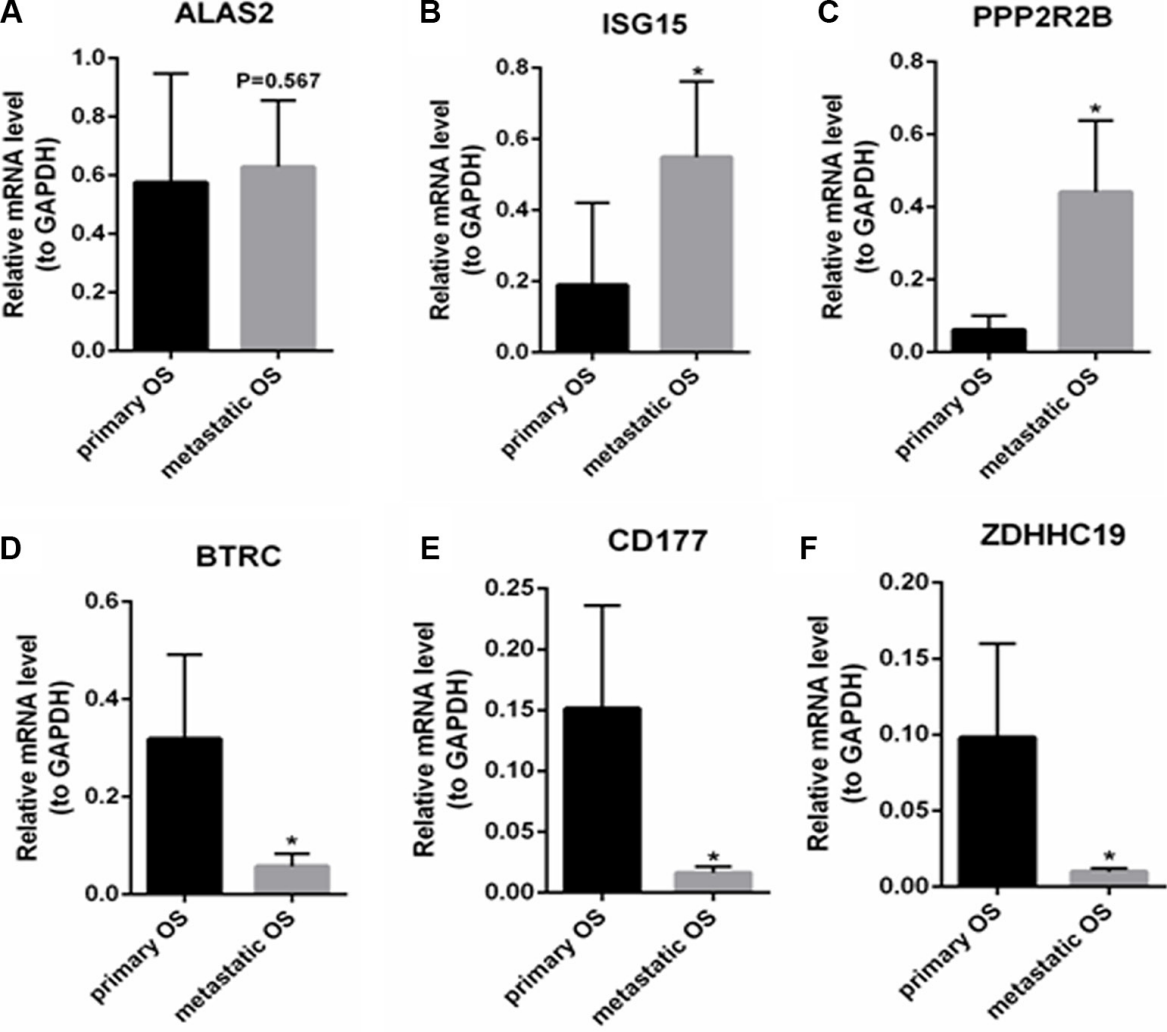
The expression levels of DEGs between primary OS and metastatic OS were verified by qRT-PCR (**A**): ALAS2; (**B**): ISG15; (**C**): PPP2R2B; (**D**): BTRC; (**E**): CD177; (**F**) ZDHHC19. Primary OS represents primary osteosarcoma and metastatic OS represents metastatic osteosarcoma. At least three independent experiments were performed for statistical evaluation.

The expression levels of DEGs between 19 tumor samples of primary OS and 19 tumor samples of metastatic OS were verified by qRT-PCR. As shown in Figure 4A, the expression level of ALAS2 had no significant difference between metastatic OS and primary OS but showed a tendency for up-regulation in metastatic OS. In Figure 4B and 4C, ISG15 and PPP2R2B were significantly up-regulated in metastatic OS compared with primary OS (*P* < 0.05). In Figure 4D, and 4F, BTRC, CD177 and ZDHHC19 were significantly down-regulated in metastatic OS compared with primary OS (*P* < 0.05). The qRT-PCR results between primary OS and metastatic OS verified our RNA-seq data.

## DISCUSSION

AURKB is overexpressed in OS tissues and cells and functions as an oncogene in OS cells [11]. Up-regulation of AURKB in Epstein-Barr virus- (EBV)-transformed lymphoblasts is correlated with EBV immortalization and tumorigenic ability *in vitro* [12]. A number of studies discover that high expression of AURKB is associated with unfavorable prognosis of cancers such as acute myeloid leukemia, nasopharyngeal carcinoma, colorectal adenocarcinoma and gastric cancer [13–16]. Suppression of AURKB down-regulates phosphorylation of VCP and activates the NF-κB signaling pathway in OS cells, resulting in inhibition of cell proliferation, migration and invasion [17]. In addition, inhibition of Aurora-B suppresses the migratory and invasive abilities of OS cells through modulating the PI3K/Akt/NF-κB signaling pathway *in vitro* [18, 19].

Protein phosphatase 2 is a member of four major Ser/Thr phosphatases and functions as negative control of cell growth and division [20]. In nasopharyngeal carcinoma, reduced PPP2R2B expression activates PDK1/MYC pathways to induce BEZ235 resistance [21]. In breast cancer patients, a variant of the rs319217 SNP in PPP2R2B is associated with a better response to chemotherapy treatment and lower risk of breast cancer recurrence [22]. In addition, up-regulated PPP2RPR is involved in the promotion of T-cell apoptosis triggered by IL-2 deficiency in systemic lupus erythematosus, an autoimmune disease [23]. The biological function of PPP2R2B in OS is unknown and further work is needed to elucidate it.

The expression of ISG15 has been implicated in the induction of type I IFN expression and the activation of NK cells, which are important mediators of tumor immunity [24]. Over-expression of ISG15 is involved in tumorigenesis and metastasis of various cancers, such as hepatocellular carcinoma, ovarian serous carcinoma, and breast cancer [25–27]. The high expression level of ISG15 has a significant correlation with unfavorable prognosis in esophageal squamous cell cancers and breast cancer [25, 28]. ISG15 is highly expressed in hepatocellular carcinoma (HCC) tumor specimens and triggers tumorigenesis and metastasis of HCC [26]. Knocking down ISG15 inhibits HCC cell proliferation and migration, arrests the cell cycle at the G2/M phase *in vitro*, and inhibits tumor growth *in vivo* [26]. Moreover, ISG15 suppresses RANKL-induced osteoclastogenic differentiation of murine RAW264 cells [29]. This is the first study to report that ISG15 is dysregulated in OS; however, the biological function of ISG15 in OS is still unclear and needs to be further elucidated through *in vivo* and *in vitro* studies.

BTRC targets NFKBIA (nuclear factor of kappa light polypeptide gene enhancer in B-cells inhibitor, alpha) for degradation and activates nuclear factor kappa-B, which is involved in innate immunity. Suppressed BTRC/ FBXW11 markedly reduces IL-17 induced degradation of ACT1, which contributes to persistent immune response in inflammatory diseases [30]. Down-regulated BTRC is associated with poor prognosis in patients with nasopharyngeal carcinoma (NPC) [31]. EBV-miR-BART10-3p negatively targets BTRC to promote cell invasion and cell migration and to facilitate the epithelial-mesenchymal transition of NPC [31].

Arachidonic acid metabolism, malaria, and chemokine signaling pathways showed the highest enrichment of DEG in metastatic OS compared to primary OS. Arachidonic acid metabolism, a key inflammatory pathway, generates peroxides, free organic radicals, and aldehydes that promote tumorigenesis in diverse cancers, such as pancreatic cancer, prostate cancer, and head and neck cancer [32–34]. Cyclooxygenase-2 (COX-2) and 5-lipoxygenase (5-LOX) form eicosanoids, which are the substrates for arachidonic acid metabolism. When COX-2 and 5-LOX are blocked, cancer cell proliferation is abrogated in non-small cell lung cancer [35]. Both COX-2 and 5-LOX are up-regulated in pancreatic cancer cell lines at the mRNA and protein levels [32]. Somatic mutations in arachidonic acid metabolism pathway genes, such as PLA2G3, PTGIS and GGT7, prolong post-treatment disease-free survival of patients with oral cancer [33]. It is reported that the change of chemokine signaling pathway is implicated in diverse cancers such as breast, lung, and colorectal cancer [36–38]. XCR1, enriched in chemokine signaling pathway, was significantly down-regulated in primary OS compared with metastasis OS (Table 3). XCR1 enhances cell growth and migration and is involved in bone metastasis in non-small cell lung cancer [39]. Based on the aforementioned information, abnormally expressed mRNAs might play vital roles in OS tumorigenesis through regulating enriched KEGG pathways.

In conclusion, we identified 111 DEGs and 235 DEGs between primary OS and normal controls and between metastatic OS and primary OS, respectively. The potential functions of dysregulated genes in primary OS and metastasis OS were predicted through GO and KEGG enrichment. The expression levels of dysregulated candidate genes were detected in OS tissues through qRT-PCR. There are limitations in our study. The biological functions of key DEGs, including AURKB, CD177, ZDHHC19, PPP2R2B, ISG15, and BTRC, in the pathophysiology of OS is unclear and might be explored through *in vitro* and *in vivo* experiments in future work. Our findings might provide a foundation for the further elucidation of tumorigenic and metastatic mechanisms of OS.

## MATERIALS AND METHODS

### Sample isolation and characterization

Twenty subjects were enrolled into our study from the Third Affiliated Hospital of Kunming Medical University, which consisted of 5 subjects with primary OS (without metastasis), 5 subjects with metastatic OS and 10 healthy controls. The human subject study was approved by the Third Affiliated Hospital of Kunming Medical University and informed written consent was obtained from all patients. 10 ml peripheral blood was obtained from each of the subjects, peripheral blood mononuclear cells (PBMCs) were isolated and total RNA was extracted using TRIzol reagent (Invitrogen, Carlsbad, CA, USA) according to the manufacturer's instructions.

### Library preparation and RNA-seq

The Illumina Truseq RNA sample Prep Kit (Illumina, Inc., San Diego, CA, USA) was used for cDNA library preparation of PBMC samples according to the manufacturer's protocol. PolyA mRNA was extracted from the total mRNA sample and purified with polyT oligo-conjugated magnetic beads, followed by mRNA fragmentation. First-strand cDNA were transcribed using random primers, followed by second strand cDNA synthesis and end repair. The product was ligated to Illumina Truseq adaptors. After PCR amplification, the enriched cDNA libraries were sequenced using Illumina HiSeq 2500 (Illumina, Inc., San Diego, CA, USA).

### Primary analysis

Libraries from samples were developed using high-throughput RNA-sequencing. The raw image data were translated into raw FASTQ sequence data by Base Calling.

Raw RNA-Seq data were filtered using FASTxtool SeqPrep (https://github.com/jstjohn/SeqPrep) and Sickle (https://github.com/najoshi/sickle)

according to the following 3 criteria: firstly, only reads containing sequencing adaptors were used; secondly, nucleotides with a quality score < 20 were trimmed from the end of the sequence; and thirdly, reads with N rate > 10% were removed.

### Reads mapping

Clean and trimmed reads were aligned with the UCSC human reference genome (hg19) using TopHat v1.3.1 [40]. TopHat allows a maximum of two mismatches when mapping the reads to the reference genome. Aligned read files were then processed by Cufflinks v1.2.1 [41], which measures the relative expression of the genes with the normalized RNA-Seq fragment counts.

Analysis of differential expression of genes fragments per kilobase of exon per million mapped reads (FPKM) was used to determine the transcription abundance of each gene. The reference GTF annotation file used in Cufflinks was downloaded from the Ensembl database (Homo_sapiens.GRCh37.63.gtf) [42]. The expression testing was performed using paired *t*-tests. After applying Benjamini-Hochberg correction for multiple tests, the *P*-value < 0.05 and abs(log2fold change > 1) was selected as the criteria for significant differential expression.

### Functional annotation of differentially expressed genes

Gene Ontology (GO) and Kyoto Encyclopedia of Genes and Genomes (KEGG) pathway enrichment analyses were used to predict the biological function of differentially expressed genes [43, 44]. FDR < 0.05 was set as the cut-off for selecting significantly enriched functional GO terms and KEGG pathway enrichment.

### Network construction of protein-protein interaction

BioGRID, a database of predicted protein interactions [45], was used to screen interacting protein pairs based on differentially expressed genes. PPI networks of up- and down-regulated differentially expressed genes were visualized using Cytoscape software (http://cytoscape.org/) [46]. In the networks, nodes represent proteins and edges represent interactions between two proteins.

### Quantitative real-time polymerase chain reaction (qRT-PCR)

Total RNA of 14 OS samples of normal tissues, 19 OS samples without metastasis and 19

OS samples with lymph node or distant metastasis were extracted using TRIzol reagent (Invitrogen, Carlsbad,CA, USA) according to the manufacturer's instructions. The ReverTra Ace qPCR RT Master Mix Kit (TOYOBO, Shanghai, China) was used to synthesize the cDNA. qRT-PCR reactions were performed using SYBR^®^ FAST qPCR Kits (KAPA bio, Boston, MA, USA) on the LightCycler 480 (Roche Indianapolis, IN, USA). GAPDH was used as internal control for mRNA detection. The relative expression of target genes was calculated using the 2^-ΔΔCT^ equation [47]. The PCR primers used are shown in Table 4. The GraphPad Prism version 6.0 software package (GraphPad Software, San Diego, CA, USA) was used to output figures. The mean ± standard deviation and independent-samples *t*-test were used in the statistical analyses. *P* < 0.05 was considered statistically significant.

**Table 4:** The primer list of genes

Genes	Gene ID	Primer sequence	
AURKB	9212	Forward Sequence:	GGAGTGCTTTGCTATGAGCTGC
		Reverse Sequence:	GAGCAGTTTGGAGATGAGGTCC
CD177	57126	Forward Sequence:	CAACCTGCTCAATGGGACACAG
		Reverse Sequence:	CTTGAGCCAAGTTTCCGTGTGTC
ZDHHC19	131540	Forward Sequence:	GCAATGGTGTCCAAAGTGCTGC
		Reverse Sequence:	AAGTTGCGGTGACCGATGCAGT
SMN1	6606	Forward Sequence:	ACCACACCTAAAAGAAAACCTGCT
		Reverse Sequence:	CCGTCTTCTGACCAAATGGCAG
CKMT1	1159	Forward Sequence:	CTGGTGACGAGGAGTCCTATGA
		Reverse Sequence:	TCCGTTGTGTGCTTCATCACCC
CEACAM8	1088	Forward Sequence:	GCGAGTGCAAACTTCAGTGACC
		Reverse Sequence:	ACTGTGAGGGTGGATTAGAGGC
PPP2R2B	5521	Forward Sequence:	ATGACTACCTCCGCAGCAAGCT
		Reverse Sequence:	CATCACGCTTGGTGTTTCTGTCG
ISG15	9636	Forward Sequence:	CTCTGAGCATCCTGGTGAGGAA
		Reverse Sequence:	AAGGTCAGCCAGAACAGGTCGT
ALAS2	212	Forward Sequence:	GCCTCAAAGGATGTGTCCGTCT
		Reverse Sequence:	TACTGGTGCCTGAGATGTTGCG
BTRC	8945	Forward Sequence:	GGACACAAACGAGGCATTGCCT
		Reverse Sequence:	CAACGCACCAATTCCTCATGGC

### SUPPLEMENTARY MATERIALS FIGURES AND TABLES










