# Supplementary material for: Identification of differentially expressed genes in the development of osteosarcoma using RNA-seq
Source: Oncotarget. 2016 Nov 24;7(52):87194–205. doi: 10.18632/oncotarget.13554 (PMC5349981; doi:10.18632/oncotarget.13554)
Supplement: Supplementary file 1 [file oncotarget-07-87194-s001.pdf]

Identification of differentially expressed genes in the development of osteosarcoma using RNA-seq

Supplementary Materials

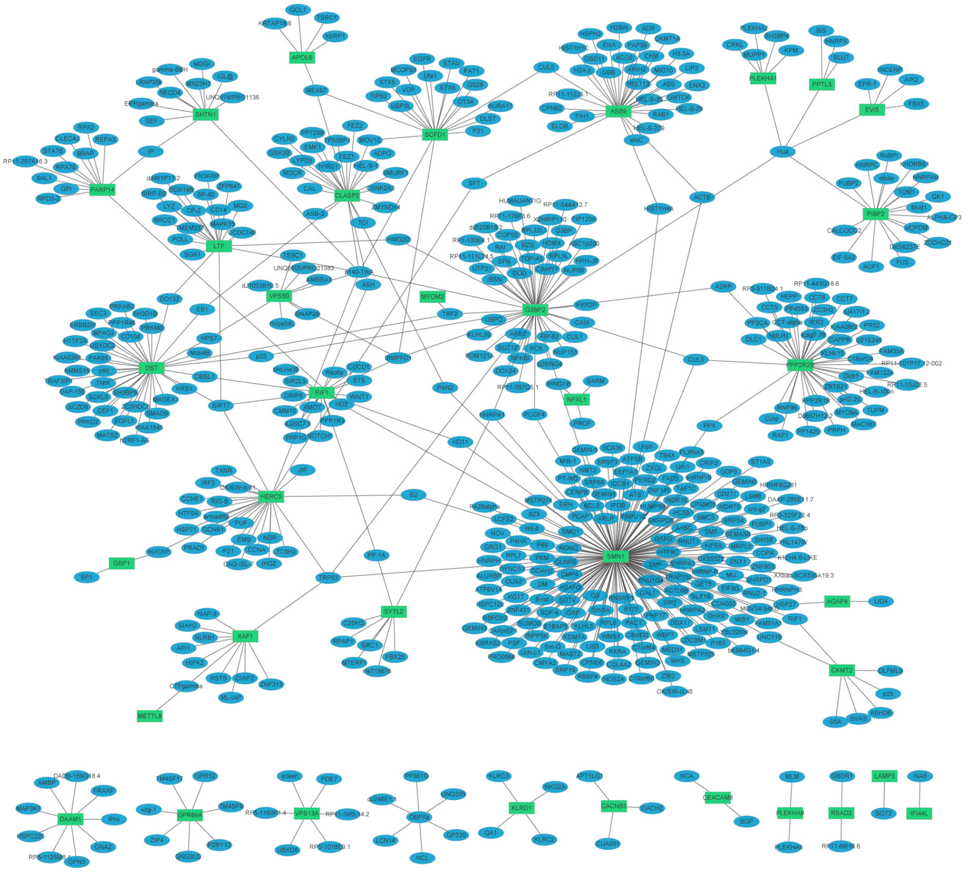

Supplementary Figure S1: The constructed PPI network of down-regulated DEGs between primary OS and normal control. The green nodes represent down-regulated DEGs and the blue nodes denote gene products predicted to interact with the DEGs.

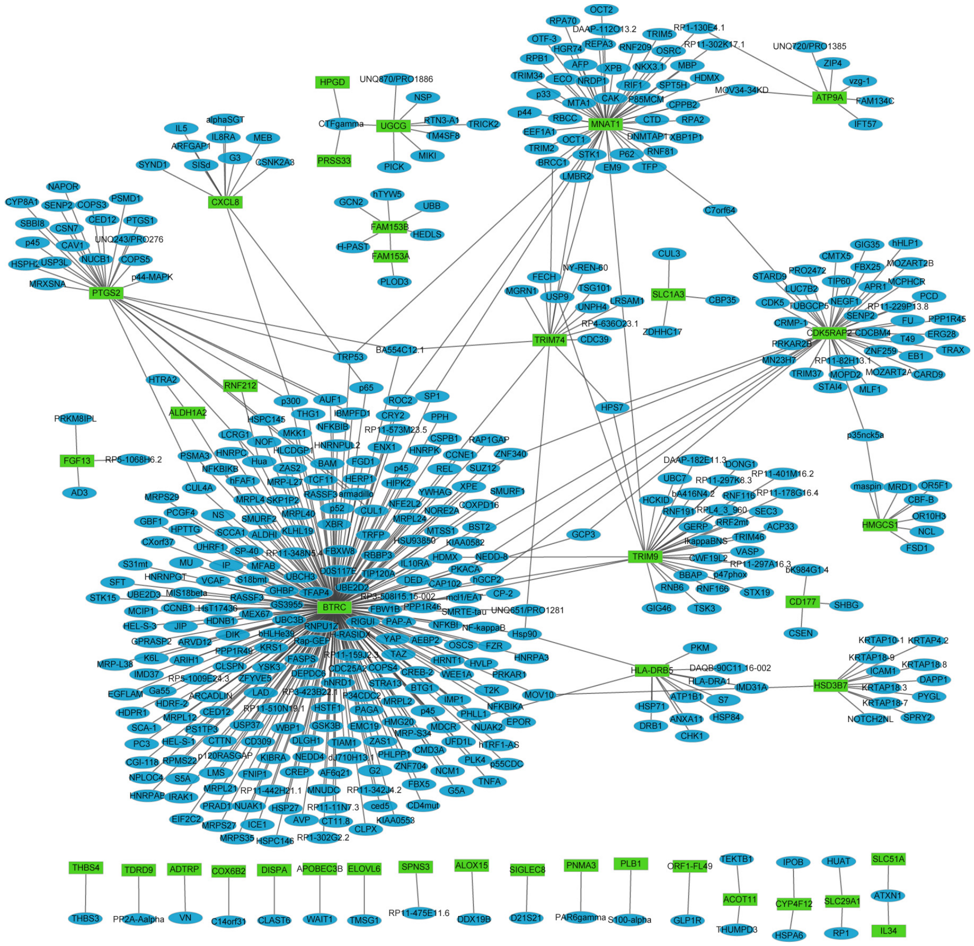

Supplementary Figure S2: The constructed PPI network of the top 50 down-regulated DEGs between metastatic OS and primary OS. The green nodes represent down-regulated DEGs and the blue nodes denote gene products predicted to interact with the DEGs.

Supplementary Table S1: The basic infromation of samples for patients in RNA-seq

No.	Gender	Age	Location of OS	MSTS stage
**Primary OS**				
P1	Male	24	Right humerus	G2T1M0,IIA
P2	Female	13	Right femur	G2T1M0,IIA
P3	Female	13	Left femur	G2T2M0,IIB
P4	Male	22	Right scapula	G2T2M0,IIB
P5	Male	16	Right tibia	G2T2M0,IIB
**Metastatic OS**				
M1	Male	17	Right foot radius	G2T1M1,III
M2	Male	22	Left femur	G2T2M1 ,III
M3	Male	56	Left femur	G2T2M1 ,III
M4	Female	17	Right femur	G2T2M1 ,III
M5	Male	17	Left femur	G2T2M1, III

No.= number; OS=osteosarcoma; MSTS = Musculoskeletal Tumor Society;P = primary OS; M = metastatic OS.

Supplementary Table S2: DEGs between primary osteosarcoma and normal control.
see Supplementary_Table_S2

Supplementary Table S3: GO terms analysis of DEGs between primary osteosarcoma and normal control (top 15).
see Supplementary_Table_S3

Supplementary Table S4: DEGs between primary osteosarcoma and metastatic osteosarcoma.
see Supplementary_Table_S4

Supplementary Table S5: GO terms analysis of DEGs between primary osteosarcoma and metastatic osteosarcoma (top 15). see Supplementary_Table_S5

Supplementary Table S6: KEGG analysis of DEGs between primary osteosarcoma and metastatic osteosarcoma (top 15)

ID	KEGG terms	FDR	Genes
hsa00590	Arachidonic acid metabolism	0.000169	AKR1C3,PLB1,ALOX15,GGT1,EPHX2,PTGS2
hsa05144	Malaria	0.000188	HBD,SDC4,KLRK1,THBS4,SELP,CD36
hsa04062	Chemokine signaling pathway	0.000662	CCR2,STAT2,CXCL5,CXCL1,ADCY3,XCR1, VAV2,PTK2,PF4V1
hsa04512	ECM-receptor interaction	0.000956	SDC4,THBS4,CD36
hsa04145	Phagosome	0.001154	THBS4,ITGB1,CD36
hsa04514	Cell adhesion molecules (CAMs)	0.001175	SDC4,SIGLEC1,HLA-DRB5,MPZL1,CD86, SELP,ITGB1
hsa04060	Cytokine-cytokine receptor interaction	0.001506	CCR2,CXCL5,CXCL1,XCR1,PF4V1
hsa05200	Pathways in cancer	0.003715	E2F1,FZD1,FGF13,EGF,PLCG1,MITF,PTK2, ITGB1,PAX8,PTGS2
hsa05150	Staphylococcus aureus infection	0.003891	C1QA,HLA-DRB5,SELP,C1QB
hsa05160	Hepatitis C	0.004369	STAT2,OAS2,EGF,OAS3,OAS1,LDLR
hsa05322	Systemic lupus erythematosus	0.004431	C1QA,HLA-DRB5,CD86,C1QB,ELANE
hsa05162	Measles	0.004592	STAT2,OAS2,OAS3,OAS1
hsa04360	Axon guidance	0.004656	ROBO3,SEMA6C,SEMA7A,PLXNA3,PTK2,ITGB1
hsa05322	Systemic lupus erythematosus	0.004723	C1QA,HLA-DRB5,C1QB
hsa04670	Leukocyte transendothelial migration	0.004988	PLCG1,PTK2,ITGB1

FDR: false discovery rate.
