# Supplementary material for: Identification of differentially expressed genes in the development of osteosarcoma using RNA-seq
Source: Oncotarget. 2016 Nov 24;7(52):87194–205. doi: 10.18632/oncotarget.13554 (PMC5349981; doi:10.18632/oncotarget.13554)
Supplement: Supplementary file 2 [file oncotarget-07-87194-s002.docx]

**Table S2 DEGs between primary osteosarcoma and normal control**

| **Gene ID** | **Gene Symbol** | ***P*-value** | **log_2_FC** |
| --- | --- | --- | --- |
| **Up-regulation genes** | | | |
| 57126 | CD177 | 0.00365 | 7.28443 |
| 131540 | ZDHHC19 | 0.0185 | 6.988027 |
| 10926 | DBF4 | 0.0307 | 5.21743 |
| 387755 | INSC | 0.02145 | 4.575003 |
| 101928079 | LINC01057 | 0.0209 | 3.910375 |
| 56729 | RETN | 0.04225 | 3.466679 |
| 6507 | SLC1A3 | 0.0346 | 3.424351 |
| 1958 | EGR1 | 0.00105 | 3.334501 |
| 202051 | SPATA24 | 0.0277 | 3.273086 |
| 84418 | CYSTM1 | 0.0357 | 3.258481 |
| 4651 | MYO10 | 0.0489 | 3.058558 |
| 79623 | GALNT14 | 0.0489 | 3.024365 |
| 6129 | RPL7 | 0.00315 | 2.909688 |
| 50486 | G0S2 | 0.0291 | 2.894525 |
| 3622 | ING2 | 0.04375 | 2.81313 |
| 9212 | AURKB | 0.0476 | 2.702396 |
| 80270 | HSD3B7 | 0.0092 | 2.693888 |
| 55755 | CDK5RAP2 | 0.02175 | 2.652372 |
| 4929 | NR4A2 | 0.03875 | 2.520546 |
| 10079 | ATP9A | 0.0476 | 2.520222 |
| 59285 | CACNG6 | 0.0499 | 2.473261 |
| 151056 | PLB1 | 0.025 | 2.416494 |
| 3127 | HLA-DRB5 | 0.00865 | 2.383275 |
| 146547 | PRSS36 | 0.0377 | 2.38252 |
| 954 | ENTPD2 | 0.01725 | 2.369787 |
| 200931 | SLC51A | 0.0453 | 2.365646 |
| 6231 | RPS26 | 0.0264 | 2.325699 |
| 1959 | EGR2 | 0.0245 | 2.323921 |
| 10912 | GADD45G | 0.0352 | 2.290673 |
| 283951 | C16orf91 | 0.02335 | 2.268699 |
| 9828 | ARHGEF17 | 0.0374 | 2.267454 |
| 5209 | PFKFB3 | 0.02155 | 2.215986 |
| 66004 | LYNX1 | 0.0217 | 2.196253 |
| 7378 | UPP1 | 0.042 | 2.170378 |
| 100526842 | RPL17-C18orf32 | 0.01845 | 2.165189 |
| 3797 | KIF3C | 0.03255 | 2.16206 |
| 23564 | DDAH2 | 0.03905 | 2.098628 |
| 3566 | IL4R | 0.0162 | 2.078543 |
| 6144 | RPL21 | 0.0366 | 2.054512 |
| 8291 | DYSF | 0.04955 | 2.043324 |
| 54512 | EXOSC4 | 0.0377 | 1.985036 |
| 3801 | KIFC3 | 0.02305 | 1.97448 |
| 7869 | SEMA3B | 0.01755 | 1.973735 |
| 5864 | RAB3A | 0.04415 | 1.962718 |
| 1 | A1BG | 0.0451 | 1.92288 |
| 347735 | SERINC2 | 0.01955 | 1.91872 |
| 54541 | DDIT4 | 0.01455 | 1.893068 |
| 51676 | ASB2 | 0.0244 | 1.838849 |
| 23329 | TBC1D30 | 0.0387 | 1.82249 |
| 2678 | GGT1 | 0.03375 | 1.814556 |
| 79603 | CERS4 | 0.01745 | 1.772691 |
| 79746 | ECHDC3 | 0.0463 | 1.772688 |
| 64651 | CSRNP1 | 0.0243 | 1.725792 |
| 11156 | PTP4A3 | 0.025 | 1.707788 |
| 6915 | TBXA2R | 0.04465 | 1.692268 |
| 25841 | ABTB2 | 0.04125 | 1.589747 |
| 11094 | CACFD1 | 0.04955 | 1.581583 |
| 83667 | SESN2 | 0.03755 | 1.533473 |
| 8482 | SEMA7A | 0.0419 | 1.519787 |
| 165140 | OXER1 | 0.048 | 1.485322 |
| 286144 | TRIQK | 0.04615 | 1.482276 |
| 57817 | HAMP | 0.04815 | 1.461759 |
| 54997 | TESC | 0.04975 | 1.455565 |
| 28227 | PPP2R3B | 0.0373 | 1.42548 |
| 8427 | ZNF282 | 0.03605 | 1.38542 |
| 2242 | FES | 0.0474 | 1.328806 |
| 387778 | SPDYC | 0.00585 | 1.221753 |
| **Down-regulation genes** | | | |
| 1160 | CKMT2 | 0.01115 | -4.28207 |
| 1088 | CEACAM8 | 0.01075 | -3.24111 |
| 10964 | IFI44L | 0.00465 | -3.14574 |
| 140462 | ASB9 | 0.0327 | -2.73412 |
| 91543 | RSAD2 | 0.0133 | -2.60049 |
| 653519 | GPR89A | 0.0213 | -2.5051 |
| 55183 | RIF1 | 0.04975 | -2.49066 |
| 7813 | EVI5 | 0.02925 | -2.41565 |
| 84725 | PLEKHA8 | 0.02415 | -2.39095 |
| 4057 | LTF | 0.0033 | -2.30934 |
| 79828 | METTL8 | 0.02985 | -2.28365 |
| 79750 | ZNF385D | 0.02565 | -2.19376 |
| 653464 | SRGAP2C | 0.0349 | -2.10654 |
| 643699 | GOLGA8N | 0.04365 | -2.10211 |
| 23230 | VPS13A | 0.0419 | -2.07423 |
| 23002 | DAAM1 | 0.043 | -2.0566 |
| 27074 | LAMP3 | 0.0324 | -2.05076 |
| 53938 | PPIL3 | 0.0252 | -2.0262 |
| 58155 | PTBP2 | 0.03495 | -2.01801 |
| 55230 | USP40 | 0.04485 | -1.99248 |
| 6606 | SMN1 | 0.0401 | -1.94627 |
| 7637 | ZNF84 | 0.04385 | -1.92633 |
| 23256 | SCFD1 | 0.01415 | -1.92168 |
| 51191 | HERC5 | 0.01015 | -1.91764 |
| 10561 | IFI44 | 0.0266 | -1.90879 |
| 54843 | SYTL2 | 0.00355 | -1.88425 |
| 9172 | MYOM2 | 0.02515 | -1.84017 |
| 119016 | AGAP4 | 0.0439 | -1.79252 |
| 667 | DST | 0.0109 | -1.77476 |
| 57698 | SHTN1 | 0.0396 | -1.75564 |
| 59338 | PLEKHA1 | 0.0332 | -1.72407 |
| 5521 | PPP2R2B | 0.01425 | -1.7237 |
| 54739 | XAF1 | 0.01875 | -1.6667 |
| 23122 | CLASP2 | 0.04345 | -1.65318 |
| 55610 | VPS50 | 0.0491 | -1.60102 |
| 6614 | SIGLEC1 | 0.0473 | -1.56536 |
| 152518 | NFXL1 | 0.03935 | -1.51968 |
| 9908 | G3BP2 | 0.04385 | -1.47247 |
| 54625 | PARP14 | 0.04775 | -1.45799 |
| 2633 | GBP1 | 0.03645 | -1.43779 |
| 784 | CACNB3 | 0.0279 | -1.32761 |
| 80830 | APOL6 | 0.0432 | -1.32628 |
| 3824 | KLRD1 | 0.03735 | -1.29792 |

FC: fold change
