# Supplementary material for: Identification of differentially expressed genes in the development of osteosarcoma using RNA-seq
Source: Oncotarget. 2016 Nov 24;7(52):87194–205. doi: 10.18632/oncotarget.13554 (PMC5349981; doi:10.18632/oncotarget.13554)
Supplement: Supplementary file 3 [file oncotarget-07-87194-s003.docx]

**Table S3 GO terms analysis of DEGs between primary osteosarcoma and normal control (top 15)**

| **GO ID** | **GO term** | **Count** | **FDR** |
| --- | --- | --- | --- |
| **Biological Process** | |  |  |
| GO:0031110 | regulation of microtubule polymerization or depolymerization | 2 | 1.53E-02 |
| GO:0009791 | post-embryonic development | 4 | 2.50E-02 |
| GO:0030278 | regulation of ossification | 2 | 2.85E-02 |
| GO:0048705 | skeletal system morphogenesis | 2 | 3.83E-02 |
| GO:0050727 | regulation of inflammatory response | 2 | 3.83E-02 |
| GO:0050975 | sensory perception of touch | 1 | 4.03E-02 |
| GO:0031630 | regulation of synaptic vesicle fusion to presynaptic membrane | 1 | 4.03E-02 |
| GO:0048790 | maintenance of presynaptic active zone structure | 1 | 4.03E-02 |
| GO:0072303 | positive regulation of glomerular metanephric mesangial cell proliferation | 1 | 4.03E-02 |
| GO:0072110 | glomerular mesangial cell proliferation | 1 | 4.03E-02 |
| GO:0033233 | regulation of protein sumoylation | 1 | 4.03E-02 |
| GO:0009449 | gamma-aminobutyric acid biosynthetic process | 1 | 4.03E-02 |
| GO:0021660 | rhombomere 3 formation | 1 | 4.03E-02 |
| GO:0021666 | rhombomere 5 formation | 1 | 4.03E-02 |
| GO:0035771 | interleukin-4-mediated signaling pathway | 1 | 4.53E-02 |
| **Molecular Function** | |  |  |
| GO:0005515 | protein binding | 26 | 3.95E-02 |
| GO:0051010 | microtubule plus-end binding | 2 | 3.97E-02 |
| GO:0005245 | voltage-gated calcium channel activity | 2 | 4.74E-02 |
| GO:0004913 | interleukin-4 receptor activity | 1 | 4.80E-02 |
| GO:0004850 | uridine phosphorylase activity | 1 | 4.80E-02 |
| GO:0005314 | high-affinity glutamate transmembrane transporter activity | 1 | 4.80E-02 |
| GO:0004727 | prenylated protein tyrosine phosphatase activity | 1 | 4.80E-02 |
| GO:0034987 | immunoglobulin receptor binding | 1 | 4.80E-02 |
| GO:0016403 | dimethylargininase activity | 1 | 4.80E-02 |
| GO:0016813 | hydrolase activity, acting on carbon-nitrogen (but not peptide) bonds, in linear amidines | 1 | 4.80E-02 |
| GO:0047016 | cholest-5-ene-3-beta,7-alpha-diol 3-beta-dehydrogenase activity | 1 | 4.81E-02 |
| GO:0050646 | 5-oxo-6E,8Z,11Z,14Z-icosatetraenoic acid binding | 1 | 4.81E-02 |
| GO:0050647 | 5-hydroxy-6E,8Z,11Z,14Z-icosatetraenoic acid binding | 1 | 4.81E-02 |
| GO:0050648 | 5(S)-hydroxyperoxy-6E,8Z,11Z,14Z-icosatetraenoic acid binding | 1 | 4.81E-02 |
| GO:0051535 | syntaxin-5 binding | 1 | 4.81E-02 |
| **Cellular Component** | |  |  |
| GO:0005737 | cytoplasm | 39 | 7.21E-07 |
| GO:0005886 | plasma membrane | 25 | 1.22E-03 |
| GO:0005829 | cytosol | 18 | 1.54E-03 |
| GO:0005622 | intracellular | 16 | 4.61E-03 |
| GO:0005819 | spindle | 4 | 7.06E-03 |
| GO:0005794 | Golgi apparatus | 10 | 8.09E-03 |
| GO:0031225 | anchored to membrane | 4 | 8.19E-03 |
| GO:0005856 | cytoskeleton | 9 | 1.21E-02 |
| GO:0005634 | nucleus | 28 | 1.59E-02 |
| GO:0005871 | kinesin complex | 2 | 1.78E-02 |
| GO:0005891 | voltage-gated calcium channel complex | 2 | 2.37E-02 |
| GO:0016021 | integral to membrane | 23 | 3.07E-02 |

FDR: false discovery rate
