# Supplementary material for: Identification of differentially expressed genes in the development of osteosarcoma using RNA-seq
Source: Oncotarget. 2016 Nov 24;7(52):87194–205. doi: 10.18632/oncotarget.13554 (PMC5349981; doi:10.18632/oncotarget.13554)
Supplement: Supplementary file 4 [file oncotarget-07-87194-s004.docx]

**Table S4 DEGs between primary osteosarcoma and metastatic osteosarcoma**

| **Gene ID** | **Gene Symbol** | ***P*-value** | **log_2_FC** |
| --- | --- | --- | --- |
| **Up-regulation genes** | | | |
| 212 | ALAS2 | 0.00135 | 4.412527 |
| 759 | CA1 | 5.00E-05 | 4.367439 |
| 3045 | HBD | 0.0238 | 3.778141 |
| 3048 | HBG2 | 0.0045 | 3.703257 |
| 8991 | SELENBP1 | 0.0145 | 3.531732 |
| 7262 | PHLDA2 | 0.00505 | 3.492722 |
| 1991 | ELANE | 5.00E-05 | 3.1807 |
| 4353 | MPO | 5.00E-05 | 3.001248 |
| 4680 | CEACAM6 | 6.00E-04 | 2.974953 |
| 27285 | TEKT2 | 0.0261 | 2.965083 |
| 3397 | ID1 | 4.00E-04 | 2.955801 |
| 116028 | RMI2 | 0.0043 | 2.895913 |
| 105 | ADARB2 | 0.0061 | 2.891606 |
| 1669 | DEFA4 | 5.00E-05 | 2.888638 |
| 1088 | CEACAM8 | 1.00E-04 | 2.82414 |
| 566 | AZU1 | 5.00E-05 | 2.804545 |
| 6035 | RNASE1 | 0.0039 | 2.774094 |
| 4940 | OAS3 | 0.00165 | 2.704037 |
| 6614 | SIGLEC1 | 5.00E-05 | 2.57251 |
| 4057 | LTF | 5.00E-05 | 2.475804 |
| 4939 | OAS2 | 0.00605 | 2.385104 |
| 1668 | DEFA3 | 0.00595 | 2.368409 |
| 654433 | PAX8-AS1 | 0.00925 | 2.340938 |
| 7849 | PAX8 | 0.01125 | 2.308688 |
| 4938 | OAS1 | 0.00015 | 2.25263 |
| 91543 | RSAD2 | 0.00235 | 2.215207 |
| 4493 | MT1E | 0.02585 | 2.108223 |
| 10964 | IFI44L | 0.00165 | 2.085884 |
| 129607 | CMPK2 | 0.00455 | 2.084079 |
| 3486 | IGFBP3 | 0.0184 | 1.935521 |
| 653820 | FAM72B | 0.04225 | 1.904742 |
| 219537 | SMTNL1 | 0.00075 | 1.892026 |
| 9636 | ISG15 | 0.0026 | 1.862953 |
| 22895 | RPH3A | 5.00E-05 | 1.83436 |
| 3039 | HBA1 | 0.0177 | 1.82451 |
| 11274 | USP18 | 0.00015 | 1.810437 |
| 79750 | ZNF385D | 0.0012 | 1.721211 |
| 9381 | OTOF | 0.0498 | 1.706446 |
| 6385 | SDC4 | 0.01755 | 1.675554 |
| 51191 | HERC5 | 1.00E-04 | 1.663637 |
| 4061 | LY6E | 0.00625 | 1.643509 |
| 135927 | C7orf34 | 0.01505 | 1.631978 |
| 4238 | MFAP3 | 0.01435 | 1.617871 |
| 1950 | EGF | 0.0092 | 1.606555 |
| 4286 | MITF | 0.00215 | 1.601789 |
| 11326 | VSIG4 | 0.0037 | 1.59238 |
| 2537 | IFI6 | 0.00035 | 1.587568 |
| 713 | C1QB | 0.00585 | 1.585159 |
| 433 | ASGR2 | 0.0307 | 1.570026 |
| 64108 | RTP4 | 0.0029 | 1.548285 |
| 25960 | ADGRA2 | 0.0274 | 1.545745 |
| 1593 | CYP27A1 | 5.00E-05 | 1.543341 |
| 8819 | SAP30 | 0.01195 | 1.530003 |
| 671 | BPI | 0.00355 | 1.494233 |
| 10644 | IGF2BP2 | 7.00E-04 | 1.450258 |
| 94240 | EPSTI1 | 0.01465 | 1.449699 |
| 5521 | PPP2R2B | 7.00E-04 | 1.407465 |
| 5197 | PF4V1 | 0.0141 | 1.398685 |
| 6374 | CXCL5 | 0.0054 | 1.391271 |
| 4257 | MGST1 | 0.04145 | 1.388004 |
| 9468 | PCYT1B | 0.0376 | 1.375047 |
| 25893 | TRIM58 | 0.00675 | 1.371379 |
| 441307 | HRAT92 | 0.0095 | 1.368252 |
| 8638 | OASL | 0.00145 | 1.349745 |
| 283849 | EXOC3L1 | 0.0235 | 1.339052 |
| 9702 | CEP57 | 0.0372 | 1.333683 |
| 58475 | MS4A7 | 0.0459 | 1.32627 |
| 57698 | SHTN1 | 0.00775 | 1.316045 |
| 60312 | AFAP1 | 0.0041 | 1.306598 |
| 7041 | TGFB1I1 | 0.00215 | 1.304622 |
| 26509 | MYOF | 0.0063 | 1.286329 |
| 10561 | IFI44 | 0.0373 | 1.283487 |
| 5954 | RCN1 | 0.0117 | 1.274607 |
| 9886 | RHOBTB1 | 0.02705 | 1.27419 |
| 653464 | SRGAP2C | 0.01875 | 1.25209 |
| 8321 | FZD1 | 0.00395 | 1.243805 |
| 116071 | BATF2 | 0.00335 | 1.243664 |
| 338773 | TMEM119 | 0.0123 | 1.242392 |
| 710 | SERPING1 | 0.0019 | 1.235675 |
| 642946 | FLVCR1-AS1 | 0.03045 | 1.220116 |
| 4811 | NID1 | 0.00235 | 1.211326 |
| 6622 | SNCA | 0.0119 | 1.20865 |
| 2012 | EMP1 | 0.0138 | 1.204267 |
| 54453 | RIN2 | 0.01535 | 1.195987 |
| 4600 | MX2 | 0.04295 | 1.193234 |
| 54055 | CYP4F29P | 0.0434 | 1.19078 |
| 199731 | CADM4 | 0.00985 | 1.188077 |
| 5909 | RAP1GAP | 0.0063 | 1.175712 |
| 10053 | AP1M2 | 0.02755 | 1.1706 |
| 9935 | MAFB | 0.03405 | 1.169867 |
| 1514 | CTSL | 0.01105 | 1.154881 |
| 57648 | KIAA1522 | 0.0192 | 1.119773 |
| 79957 | PAQR6 | 0.0085 | 1.11066 |
| 130589 | GALM | 0.005 | 1.106158 |
| 6580 | SLC22A1 | 0.04385 | 1.104956 |
| 1903 | S1PR3 | 0.0468 | 1.095197 |
| 948 | CD36 | 0.0461 | 1.088994 |
| 23208 | SYT11 | 0.01705 | 1.079867 |
| 90288 | EFCAB12 | 0.0479 | 1.071051 |
| 284417 | TMEM150B | 0.0353 | 1.061894 |
| 9242 | MSC | 0.04105 | 1.038026 |
| 64135 | IFIH1 | 0.0307 | 1.031681 |
| 463 | ZFHX3 | 0.0367 | 1.025781 |
| 342510 | CD300E | 0.03135 | 1.021966 |
| 9881 | TRANK1 | 0.0304 | 1.016696 |
| 79840 | NHEJ1 | 0.03415 | 1.012225 |
| 51148 | CERCAM | 0.0107 | 1.0099 |
| 25907 | TMEM158 | 0.02845 | 1.009021 |
| 1869 | E2F1 | 0.0314 | 1.005909 |
| **Down-regulation genes** | | | |
| 131540 | ZDHHC19 | 5.00E-05 | -5.74283 |
| 57126 | CD177 | 5.00E-05 | -5.31558 |
| 79071 | ELOVL6 | 0.0075 | -3.58835 |
| 6507 | SLC1A3 | 1.00E-04 | -3.28638 |
| 2829 | XCR1 | 0.0041 | -2.88172 |
| 84976 | DISP1 | 1.00E-04 | -2.71269 |
| 1.02E+08 | LINC01057 | 0.00265 | -2.58354 |
| 3248 | HPGD | 0.00455 | -2.47742 |
| 125965 | COX6B2 | 0.01395 | -2.40323 |
| 2258 | FGF13 | 0.01515 | -2.39481 |
| 7060 | THBS4 | 0.01375 | -2.39479 |
| 8945 | BTRC | 1.00E-04 | -2.3576 |
| 146433 | IL34 | 0.0235 | -2.30004 |
| 122402 | TDRD9 | 0.0024 | -2.28361 |
| 10079 | ATP9A | 0.0023 | -2.17761 |
| 201305 | SPNS3 | 5.00E-05 | -2.13998 |
| 3576 | CXCL8 | 3.00E-04 | -2.12548 |
| 260429 | PRSS33 | 0.00035 | -2.12042 |
| 7357 | UGCG | 0.03615 | -2.00653 |
| 55755 | CDK5RAP2 | 0.00765 | -1.98234 |
| 114088 | TRIM9 | 0.0153 | -1.94339 |
| 80270 | HSD3B7 | 3.00E-04 | -1.9175 |
| 1.01E+08 | CHRM3-AS2 | 0.0012 | -1.90442 |
| 5743 | PTGS2 | 0.00705 | -1.87613 |
| 84418 | CYSTM1 | 0.0168 | -1.87336 |
| 84830 | ADTRP | 0.00065 | -1.87238 |
| 2030 | SLC29A1 | 8.00E-04 | -1.8368 |
| 23105 | FSTL4 | 0.04645 | -1.78882 |
| 246 | ALOX15 | 0.0014 | -1.76865 |
| 202134 | FAM153B | 0.00065 | -1.76124 |
| 200931 | SLC51A | 0.0185 | -1.75002 |
| 25850 | ZNF345 | 0.03405 | -1.74789 |
| 2865 | FFAR3 | 0.0324 | -1.73825 |
| 151056 | PLB1 | 0.0061 | -1.7345 |
| 4331 | MNAT1 | 0.02895 | -1.73323 |
| 55512 | SMPD3 | 0.0013 | -1.72642 |
| 1178 | CLC | 0.00025 | -1.72205 |
| 26027 | ACOT11 | 0.006 | -1.71792 |
| 285498 | RNF212 | 0.0236 | -1.691 |
| 27181 | SIGLEC8 | 0.00065 | -1.69098 |
| 1.01E+08 | LINC00665 | 0.01575 | -1.67261 |
| 8854 | ALDH1A2 | 0.04515 | -1.67109 |
| 3127 | HLA-DRB5 | 0.0212 | -1.66717 |
| 29944 | PNMA3 | 0.00215 | -1.64322 |
| 66002 | CYP4F12 | 3.00E-04 | -1.64049 |
| 9582 | APOBEC3B | 0.00245 | -1.63077 |
| 285596 | FAM153A | 0.00275 | -1.60823 |
| 3157 | HMGCS1 | 0.0166 | -1.60058 |
| 8876 | VNN1 | 0.00395 | -1.56753 |
| 378108 | TRIM74 | 0.01745 | -1.55229 |
| 55471 | NDUFAF7 | 0.01955 | -1.53777 |
| 2015 | ADGRE1 | 6.00E-04 | -1.49475 |
| 8482 | SEMA7A | 2.00E-04 | -1.49282 |
| 838 | CASP5 | 0.03985 | -1.47858 |
| 731275 | LINC01347 | 0.00395 | -1.47194 |
| 9828 | ARHGEF17 | 0.0205 | -1.45005 |
| 401466 | C8orf59 | 0.04165 | -1.44331 |
| 638 | BIK | 0.01265 | -1.43963 |
| 6231 | RPS26 | 0.0024 | -1.43499 |
| 28227 | PPP2R3B | 3.00E-04 | -1.42974 |
| 56729 | RETN | 0.00775 | -1.40628 |
| 154761 | LOC154761 | 0.01835 | -1.39431 |
| 79623 | GALNT14 | 0.04265 | -1.39058 |
| 4054 | LTBP3 | 0.011 | -1.38525 |
| 414328 | IDNK | 0.0374 | -1.38163 |
| 4651 | MYO10 | 0.04725 | -1.36177 |
| 51676 | ASB2 | 0.0035 | -1.35885 |
| 9737 | GPRASP1 | 0.01665 | -1.34724 |
| 3566 | IL4R | 0.0067 | -1.34284 |
| 5754 | PTK7 | 0.0362 | -1.33773 |
| 4671 | NAIP | 0.03945 | -1.32767 |
| 642475 | MROH6 | 0.00995 | -1.32483 |
| 5016 | OVGP1 | 0.04735 | -1.32388 |
| 4929 | NR4A2 | 0.04845 | -1.32185 |
| 11251 | PTGDR2 | 0.0195 | -1.31596 |
| 2352 | FOLR3 | 0.03955 | -1.30909 |
| 5266 | PI3 | 0.0462 | -1.29952 |
| 79608 | RIC3 | 0.01355 | -1.29904 |
| 3749 | KCNC4 | 0.02785 | -1.28112 |
| 60437 | CDH26 | 0.0388 | -1.27877 |
| 3801 | KIFC3 | 0.01065 | -1.27322 |
| 54587 | MXRA8 | 0.0181 | -1.26781 |
| 9311 | ASIC3 | 0.0169 | -1.26718 |
| 10942 | PRSS21 | 0.01675 | -1.23779 |
| 117145 | THEM4 | 0.008 | -1.22805 |
| 50512 | PODXL2 | 0.0257 | -1.20985 |
| 2590 | GALNT2 | 0.0247 | -1.20595 |
| 55007 | FAM118A | 0.0035 | -1.20193 |
| 64221 | ROBO3 | 0.00635 | -1.19243 |
| 2919 | CXCL1 | 0.0095 | -1.19126 |
| 55973 | BCAP29 | 0.02015 | -1.17483 |
| 57496 | MKL2 | 0.0446 | -1.17079 |
| 4306 | NR3C2 | 0.0262 | -1.16908 |
| 158160 | HSD17B7P2 | 0.04665 | -1.15869 |
| 284415 | VSTM1 | 0.00495 | -1.1579 |
| 3757 | KCNH2 | 0.0266 | -1.15445 |
| 57504 | MTA3 | 0.0046 | -1.14634 |
| 5208 | PFKFB2 | 0.04975 | -1.14428 |
| 1739 | DLG1 | 0.01555 | -1.14334 |
| 375449 | MAST4 | 0.01755 | -1.13361 |
| 7993 | UBXN8 | 0.03845 | -1.12076 |
| 2696 | GIPR | 0.0486 | -1.11562 |
| 9653 | HS2ST1 | 0.02855 | -1.10859 |
| 8028 | MLLT10 | 0.0439 | -1.0935 |
| 1053 | CEBPE | 0.0115 | -1.08865 |
| 259173 | ALS2CL | 0.0202 | -1.08367 |
| 1663 | DDX11 | 0.0081 | -1.07992 |
| 57124 | CD248 | 0.01495 | -1.07842 |
| 1E+08 | NPIPB5 | 0.01755 | -1.07466 |
| 4240 | MFGE8 | 0.0079 | -1.07311 |
| 84255 | SLC37A3 | 0.0289 | -1.07228 |
| 6490 | PMEL | 0.0156 | -1.0685 |
| 2678 | GGT1 | 0.0479 | -1.06509 |
| 26013 | L3MBTL1 | 0.0118 | -1.06297 |
| 2623 | GATA1 | 0.0118 | -1.05386 |
| 93134 | ZNF561 | 0.0452 | -1.05133 |
| 80221 | ACSF2 | 0.0022 | -1.04464 |
| 109 | ADCY3 | 0.02735 | -1.04058 |
| 54039 | PCBP3 | 0.0271 | -1.03335 |
| 115350 | FCRL1 | 0.04015 | -1.02961 |
| 10500 | SEMA6C | 0.0474 | -1.02853 |
| 8644 | AKR1C3 | 0.03795 | -1.02706 |
| 8481 | OFD1 | 0.03125 | -1.02616 |
| 3563 | IL3RA | 0.0146 | -1.01409 |
| 5004 | ORM1 | 0.02485 | -1.01048 |
| 9651 | PLCH2 | 0.0232 | -1.00368 |

FC: fold change
