# Supplementary material for: Identification of differentially expressed genes in the development of osteosarcoma using RNA-seq
Source: Oncotarget. 2016 Nov 24;7(52):87194–205. doi: 10.18632/oncotarget.13554 (PMC5349981; doi:10.18632/oncotarget.13554)
Supplement: Supplementary file 5 [file oncotarget-07-87194-s005.docx]

**Table S5 GO terms analysis of DEGs between primary osteosarcoma and metastatic osteosarcoma (top 15)**

| **ID** | **Terms** | **Genes** | **FDR** |
| --- | --- | --- | --- |
| **Biological process** | | | |
| GO:0019221 | cytokine-mediated signaling pathway | 15 | 7.00E-09 |
| GO:0060337 | type I interferon-mediated signaling pathway | 11 | 1.04E-08 |
| GO:0006955 | immune response | 18 | 5.59E-07 |
| GO:0006954 | inflammatory response | 14 | 2.45E-06 |
| GO:0007155 | cell adhesion | 19 | 1.41E-05 |
| GO:2000060 | positive regulation of protein ubiquitination involved in ubiquitin-dependent protein catabolic process | 3 | 1.46E-05 |
| GO:0007596 | blood coagulation | 17 | 1.57E-05 |
| GO:0006810 | transport | 5 | 4.95E-05 |
| GO:0032020 | ISG15-protein conjugation | 3 | 5.23E-05 |
| GO:0045087 | innate immune response | 13 | 5.96E-05 |
| GO:0009615 | response to virus | 9 | 7.79E-05 |
| GO:0060333 | interferon-gamma-mediated signaling pathway | 4 | 0.000163 |
| GO:0044419 | interspecies interaction between organisms | 4 | 0.000163 |
| GO:0007165 | signal transduction | 26 | 0.000176 |
| GO:0051726 | regulation of cell cycle | 6 | 0.000291 |
| **Cellular component** | | | |
| GO:0005737 | cytoplasm | 98 | 1.02E-11 |
| GO:0005886 | plasma membrane | 72 | 8.45E-10 |
| GO:0016020 | membrane | 77 | 1.69E-09 |
| GO:0005829 | cytosol | 51 | 3.51E-09 |
| GO:0016021 | integral to membrane | 76 | 1.05E-07 |
| GO:0016323 | basolateral plasma membrane | 11 | 7.52E-07 |
| GO:0005829 | cytosol | 36 | 2.25E-06 |
| GO:0005576 | extracellular region | 41 | 2.75E-06 |
| GO:0005783 | endoplasmic reticulum | 4 | 1.08E-05 |
| GO:0005739 | mitochondrion | 4 | 1.08E-05 |
| GO:0005792 | microsome | 4 | 1.08E-05 |
| GO:0005615 | extracellular space | 23 | 1.15E-05 |
| GO:0005634 | nucleus | 46 | 1.24E-05 |
| GO:0016323 | basolateral plasma membrane | 9 | 1.25E-05 |
| GO:0005887 | integral to plasma membrane | 26 | 2.09E-05 |
| **Molecular Component** | | | |
| GO:0005515 | protein binding | 88 | 5.16E-12 |
| GO:0046872 | metal ion binding | 54 | 4.05E-06 |
| GO:0004872 | receptor activity | 35 | 1.62E-05 |
| GO:0042803 | protein homodimerization activity | 18 | 2.67E-05 |
| GO:0005529 | sugar binding | 10 | 0.000114 |
| GO:0005524 | ATP binding | 31 | 0.000129 |
| GO:0008270 | zinc ion binding | 37 | 0.000132 |
| GO:0046872 | metal ion binding | 20 | 0.000251 |
| GO:0005509 | calcium ion binding | 18 | 0.000346 |
| GO:0003824 | catalytic activity | 13 | 0.000346 |
| GO:0000166 | nucleotide binding | 5 | 0.000354 |
| GO:0016817 | acting on acid anhydrides | 3 | 0.000362 |
| GGO:0016779 | nucleotidyltransferase activity | 3 | 0.000362 |
| GO:0020037 | heme binding | 7 | 0.000372 |
| GO:0003723 | RNA binding | 3 | 0.000449 |

FDR: false discovery rate.
